# Removal of Laryngeal Mask Airway in Adults Under Target-Controlled, Propofol–Fentanyl Infusion Anesthesia: Awake or Deep Anesthesia?

**DOI:** 10.1097/MD.0000000000003441

**Published:** 2016-04-29

**Authors:** Ren-Chih Huang, Nan-Kai Hung, Chueng-He Lu, Zhi-Fu Wu

**Affiliations:** From the Department of Anesthesiology, National Defense Medical Center and Tri-Service General Hospital, Taipei, Taiwan, Republic of China (R-CH, N-KH, C-HL); and Keelung Branch and Department of Anesthesiology, Tri-Service General Hospital and National Defense Medical Center, Taipei, Taiwan, Republic of China (Z-FW).

## Abstract

After emergence from anesthesia, the incidence and severity of adverse airway effects caused by the laryngeal mask airway (LMA) can vary, depending on when the device was removed; nonetheless, reports differ regarding the exact optimal timing of LMA removal. The purpose of this study was to compare the rate of adverse events between 2 groups: those whose LMA was removed under general anesthesia (“deep” group) or under target-controlled infusion (TCI) of propofol (“awake” group).

Institutional Review Board approval and written informed consent were obtained; 124 patients were then randomly allocated into either the “awake” group or the “deep” group. Anesthesia was induced and maintained using TCI of propofol, as well as intravenous fentanyl. In the “deep” group, the LMA was removed after surgery while the patients were deeply anesthetized using a target effect-site propofol concentration of 2 μg/mL, whereas in the “awake” group, the device was removed while the patients followed verbal instructions. The incidence of the following adverse events was recorded: coughing, straining, bronchospasm, laryngospasm, clenching, breath holding, gross purposeful movement, airway obstruction, retching, vomiting, and oxygen desaturation. If any such event occurred, the LMA removal was considered a failure. Airway hyperreactivity was recorded and graded – based on the severity of cough, breath holding, and oxygen desaturation.

The failure rate was higher in the “awake” group (15/61; 24.6%) than in the “deep” group (5/60; 8.3%). Airway hyperreactivity was mild (score, <3) in both groups.

Removal of the LMA under deep anesthesia using a target-controlled, effect-site propofol concentration of 2 μg/mL may be safer and more successful than removal when patients are fully awake after surgery.

## INTRODUCTION

The laryngeal mask airway (LMA) is one of the most popular airway devices used to keep the airway open during anesthesia. The main advantages of the LMA over the endotracheal tube are its rapid and easy placement, hemodynamic stability at induction, smooth emergence from anesthesia, and low associated incidence of sore throat.^1^

The instruction manual suggests that the LMA can be safely removed when the patient has regained consciousness or protective laryngopharyngeal reflexes.^2^ However, some studies have reported that when the LMA is removed in patients under volatile or propofol anesthesia, fewer airway complications occur than when patients are awake for the removal.^3–7^ In this way, it is currently unclear whether the LMA should be removed at the end of the surgical procedure while the patient remains anesthetized (deep removal) or after the patient is fully awake (awake removal).^8^ The quality of evidence available in this regard is considered “low” or “very low;” therefore, well-designed, randomized controlled trials are warranted to demonstrate whether early removal of the LMA after general anesthesia (GA) is better than late removal.

Propofol is widely used during LMA insertion and provides a satisfactory effect; for instance, there is less cardiorespiratory depression when anesthesia is induced using propofol or target-controlled infusion (TCI) of propofol.^9–12^ In addition, the choice of anesthetics during induction or maintenance of anesthesia may contribute to airway reactivity, which manifests during emergence from anesthesia.^13–16^ Heidari et al^4^ compared propofol infusion anesthesia with halothane anesthesia and found that propofol infusion is associated with a lower incidence and severity of airway hyperreactivity both during and after LMA removal. Moreover, they showed that propofol infusion anesthesia, combined with LMA removal under deep anesthesia, is associated with the lowest incidences of coughing and straining, breath holding, and vomiting. Nonetheless, few studies have investigated the timing of LMA removal, which is associated with the incidence and severity of adverse airway effects after emergence from anesthesia using TCI combined with propofol.

In this study, we recruited adults undergoing elective surgeries and divided them into groups on the basis of anesthesia depth during LMA removal – anesthesia had been induced using TCI of propofol. We compared the incidence of adverse respiratory effects, as well as the severity of airway hyperreactivity, between the groups.

## METHODS

The ethics committee of the Tri-Service General Hospital (Taipei, Taiwan, Republic of China; TSGHIRB No: 100-05-168) approved this study. Based on the patients’ anesthetic state during LMA removal, we randomized them 1:1 into either the “deep” group or the “awake” group (see below) using a table of random, computer-generated digits in sealed, and numbered envelopes. The exclusion criteria were as follows: active upper respiratory tract infection 2 weeks before surgery or sooner, active lower respiratory tract infection 4 weeks before surgery or sooner, airway disease (asthma and chronic obstructive pulmonary disease), airway surgery, any contraindication for LMA, pregnancy, change of LMA, and administration of any drug that affects the airway during anesthesia.

The patients were not premedicated before anesthesia was induced, and they were regularly monitored: blood pressure was noninvasively evaluated, electrocardiography (lead II) and pulse oximetry were performed, and end-tidal carbon dioxide pressure was measured. Total intravenous anesthesia (TIVA) was induced using intravenous fentanyl (1 μg/kg), and a continuous infusion of propofol (Fresfol 1%) was subsequently delivered using Schneider kinetic model of TCI (Fresenius Orchestra Primea; Fresenius Kabi AG, Bad Homburg, Germany), with an effect-site concentration of 4.0 μg/mL. In all patients, the LMA was inserted by an experienced anesthesiologist according to the manufacturer's recommendations.^17^ In brief, the prescribed technique was used to choose the size and cuff inflation volume of the LMA, as well as to insert the device.^2^ The trapezius was squeezed, and if no reaction was observed, placement of the LMA was carefully attempted. If the jaw was inadequately relaxed, or if the patient coughed and swallowed during LMA insertion, the maneuver was terminated, and the propofol concentration was increased by 0.5 μg/mL. After a new equilibrium had been reached between the plasma and effect-site calculated concentration of propofol, the LMA insertion was attempted again. This sequence was repeated until the LMA had been successfully placed.

Anesthesia was maintained using TCI with a propofol concentration of 3 to 4 μg/mL, and spontaneous breathing was maintained under an oxygen flow of 0.3 L/min. Repetitive bolus injections of fentanyl were administered as required throughout the procedure. The propofol concentration was adjusted in accordance with hemodynamics, 0.2 μg/mL at a time. If 2 increments or decrements were unsuccessful, this was increased to 0.5 μg/mL.

At the end of surgery, propofol was discontinued, and 100% oxygen was administered. In the “awake” group, when the patient was awake (spontaneous eye opening, purposeful movement of the extremities without any physical stimulation, and response to verbal commands), the LMA was removed. In the “deep” group, the LMA was removed when the propofol concentration was lower than 2.0 μg/mL. The patients’ heads were in the left lateral position, and they received 100% oxygen via a face mask, without a ventilation assistant, until they were completely awake. After LMA removal, the patients were sent to the postanesthesia care unit.

When patients developed coughing, clenched teeth, retching, vomiting, or gross purposeful movements during or within 1 minute of LMA removal, or breath holding, laryngospasm, bronchospasm, airway obstruction, or desaturation to an SpO_2_ of <90% during or immediately after LMA removal, the maneuver was considered unsuccessful. Additionally, airway hyperreactivity scores^18^ were recorded. The severity of airway hyperreactivity was graded as mild (≤3), moderate (4–8), and severe (≥9). A more severe form of airway complication, characterized by airway obstruction and an SpO_2_ of <85% for more than 10 seconds, was termed a critical airway event.

Before beginning this study, we performed a power calculation to determine the ideal sample size. A minimum of 60 patients in each group was required to detect a reduction from 21%^4^ to 16% in the incidence of unsuccessful LMA removal with a power of 80% and a confidence interval of 95%. This sample size estimation was performed using GraphPad StatMate version 2.00 (GraphPad Software, Inc. 7825 Fay Avenue, Suite 230 La Jolla, CA 92037 USA). To allow for potential dropouts, we enrolled a total of 62 patients in each group. The data are presented as mean ± standard deviation (SD), number of patients, or percentage. Demographic data were analyzed using analysis of variance (ANOVA). Study variables were analyzed using the Chi-square test (Fisher exact test), and data regarding the severity of hyperreactivity were analyzed using the Kruskal–Wallis and Mann–Whitney *U* tests. A *P*-value <0.05 was considered statistically significant.

## RESULTS

**FIGURE 1:**
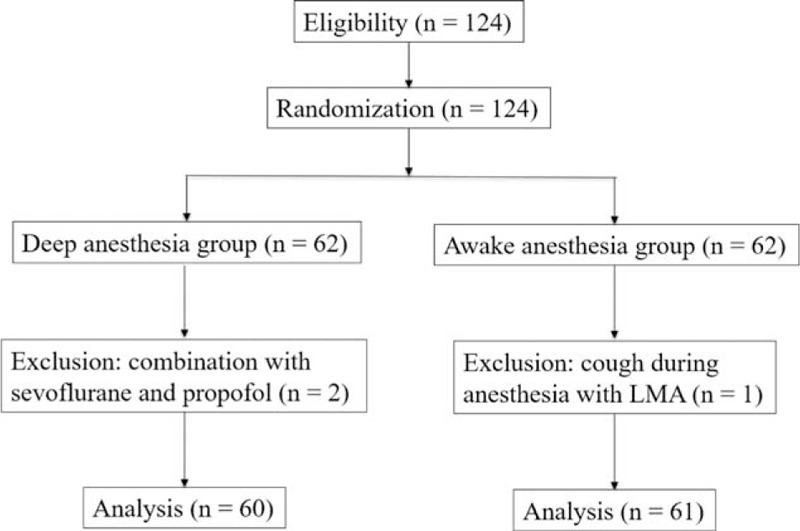
Flow diagram showing patient flow according to the study protocol.

The LMA was successfully inserted in 124 patients undergoing elective surgeries under TCI with propofol. One case in the “awake” group was excluded because of coughing during anesthesia. Two cases in the “deep” group were excluded because sevoflurane had been added to the TCI propofol anesthetic regime due to movement during anesthesia that affected the surgery. Ultimately, 121 patients completed the study: 60 in the “deep” group and 61 in the “awake” group (Figure 1). The groups showed similar patient characteristics (Table 1), and there was no significant difference between the groups in terms of operation and anesthesia time, total consumption of propofol and fentanyl, and the awakening effect-site concentration of propofol (*P* = 0.11). The failure rates after LMA removal were 24.6% and 8.3% in the “awake” and “deep” groups, respectively (*P* = 0.03; Table 2), while the overall failure rate of LMA removal during propofol anesthesia was 16.5%. Table 3 details the numbers of each unsuccessful trial during and after LMA removal. In the “deep” group, 4 patients showed desaturation, 2 displayed clenching, 1 had gross purposeful movement, 4 exhibited breath holding, and 1 had airway obstruction. In the “awake” group, 5 patients had cough, 1 exhibited desaturation, 6 showed clenching, and 7 displayed gross purposeful movement. After LMA removal, airway hyperreactivity scores did not differ significantly between the groups (*P* = 0.55). There were 6 (9.84%) and 5 (8.33%) patients with airway hyperreactivity in the “awake” and “deep” groups, respectively (*P* = 0.98; Table 2); all had scores of <3 in both groups.

**TABLE 1:**
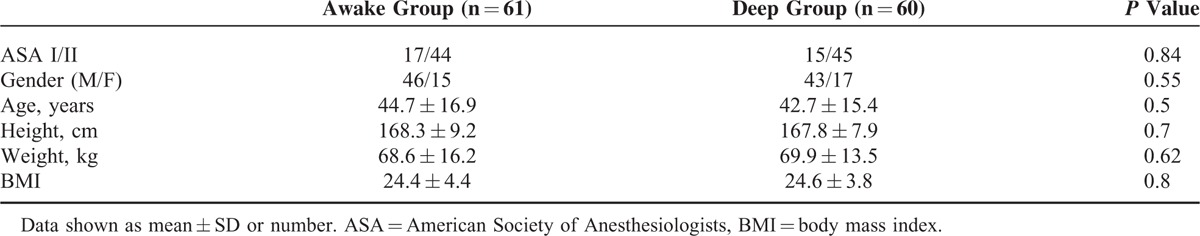
Patient Characteristics

**TABLE 2:**
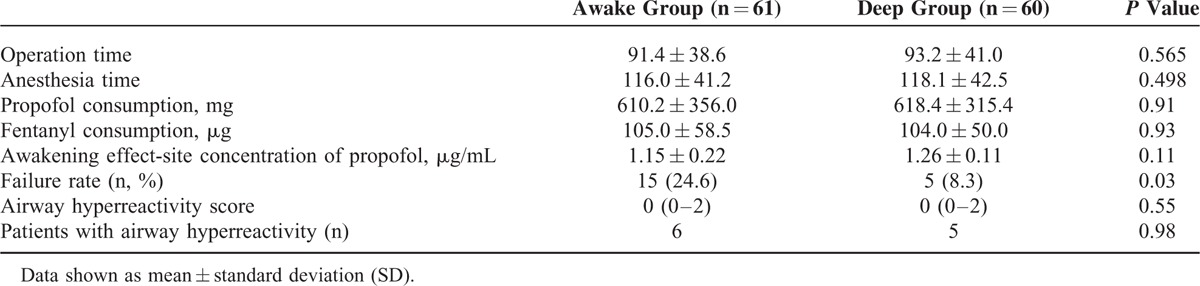
Comparison of Perioperative Characteristics and Outcomes for the 2 Groups

**TABLE 3:**
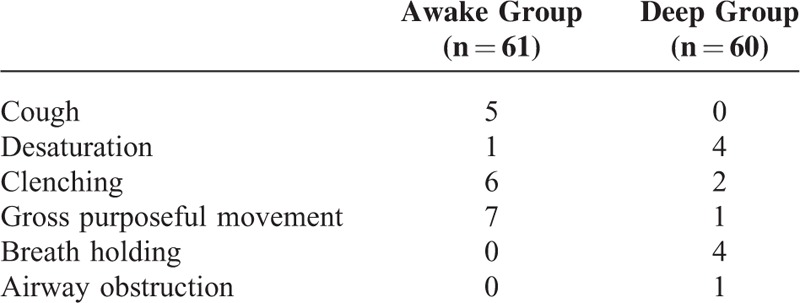
The Manifestation of Unsuccessful Removal of Laryngeal Mask Airway (LMA) in the 2 Groups

## DISCUSSION

The major finding of this study was that the failure rate of LMA removal during emergence was lower in the “deep” group, at an estimated propofol concentration of 2.0 μg/mL, than in the “awake” group. In addition, we found that airway hyperreactivity was mild under TIVA with propofol.

Investigators differ regarding the exact optimal timing of LMA removal. In fact, it varies depending on the depth of anesthesia (awake or deep) and choice of anesthetic drug (violate or propofol) – the time required for LMA removal under violate anesthesia has been widely investigated. During sevoflurane anesthesia, the LMA can be safely removed at an approximate minimum alveolar concentration of 0.86 in 95% of anesthetized children;^19^ the EC_95_ in anesthetized adults is an end-tidal sevoflurane concentration of 1.18%,^20^ and that of end-tidal desflurane to allow smooth LMA removal is 3.9% in adults.^21^ However, according to a recent systematic review, the current best evidence is inconclusive regarding whether the LMA should be removed early or late in patients undergoing GA.^8^ They concluded that there was a smaller risk of coughing after early removal (13.9%) than after late removal (19.4%), and the risk of airway obstruction was higher with early removal (15.6%) than with late removal (4.6%). In addition, there was no difference in the risk of desaturation between early removal (7.9%) and late removal (10.1%), and laryngospasm occurred at similar rates (early removal: 3.3%, late removal: 2.7%). Our results corroborated this previous report; however, the review did not enroll any studies associated with GA using propofol or TCI of propofol. Propofol is a potent inhibitor of airway reflexes at hypnotic concentrations,^22,23^ and even subhypnotic doses prevent laryngospasm during extubation in children.^24^ Indeed, Hohlrieder et al^25^ showed that, after elective lumbar disk surgery, the incidence of coughing during emergence was smaller when clinicians used propofol anesthesia with TIVA than when they used sevoflurane anesthesia. In addition, the same authors studied the influence of depth of anesthesia (“awake” or “deep”) and choice of anesthetic drug (halothane or propofol) on the incidence and severity of LMA removal-associated airway hyperreactivity. They found that, in adults, the incidence of airway hyperreactivity was higher in halothane anesthesia than in propofol. They also reported that the failure rates in the “deep” and “awake” groups after propofol anesthesia were 7.7% and 20.5%, respectively.^4^ In the “deep” group, they maintained an infusion rate of 100 μg/kg/min during LMA removal, and the concentration of propofol (approximately 3 μg/mL) was higher than that found in the current study. However, the success rate of LMA removal was comparable with that in the current study. Taken together, these findings demonstrate a decreased incidence of coughing and straining, bronchospasm, laryngospasm, vomiting, oxygen desaturation, and severity of airway hyperreactivity when using propofol. In addition, a recent study investigated TIVA with remifentanil and propofol to facilitate emergence after the LMA removal.^26^ The investigators reported that, at an optimal remifentanil concentration of 1.35 ng/mL with TCI, and a propofol concentration of approximately 1.0 μg/mL, they achieved smooth and safe emergence in 95% of patients during an awake state. Therefore, we propose that a deeper propofol anesthesia, followed by emergence after LMA removal, is safer than an awake concentration of propofol.

To the best of our knowledge, no study has investigated the incidence and severity of airway complications after LMA removal during emergence from propofol anesthesia using a TCI system. Furthermore, no dose-finding investigation has established the concentration of propofol most effective in facilitating successful LMA removal during emergence after TCI of propofol. Therefore, our study might be the first to demonstrate the optimal concentration of TCI-administered propofol (2.0 μg/mL) that allows safe LMA removal in adults.

Some adverse effects, including mild involuntary movements and apnea, can occur during LMA anesthesia using TCI of propofol.^12^ In our study, 2 patients were excluded owing to involuntary movement that affected the surgery, and 1 owing to cough during anesthesia. No other hemodynamic or respiratory complications occurred during the TCI-administered propofol anesthesia. In our study, the unsuccessful removal contributed not only to adverse respiratory events, but also to clenching and gross purposeful movement. The incidence and severity of airway hyperreactivity during TCI-administered propofol anesthesia in the “awake” group were similar to those in the “deep” group, and they were mild in both groups.

There were several concerning limitations in this study. First, we did not use a bispectral index (BIS) to monitor level of consciousness during or after LMA removal. The BIS has been widely used to monitor the level of consciousness associated with anesthetic depth in patients undergoing surgery. However, BIS results may not reliably reduce airway complications during LMA placement.^27^ In our study, the total consumption and awakening effect-site concentration of propofol were not significantly different between the “awake” and “deep” groups. Moreover, no patients reported during the postoperative visit that they had been conscious during LMA removal with a propofol concentration of 2.0 μg/mL. A second limitation of the study is that we did not exclude smokers; smoking can, at least theoretically, increase the incidence and severity of upper airway hyperactivity. However, smokers did not differ significantly from nonsmokers in terms of incidence of coughing during emergence from propofol anesthesia.^28^ Therefore, we believe that the smoking bias can be ignored, because of the suppressive effect of propofol on the airway reflex and the normal distribution in the “deep” and “awake” groups in the present study. Third, our study was limited to short-duration surgery. Further investigations into this matter involving other types of surgeries should be conducted with caution. Fourth, in “deep” group patients, we only explored a propofol concentration of 2.0 μg/mL. A previous study involving dental anesthesia demonstrated that moderate sedation can be achieved using the TCI system and a plasma propofol concentration of 1.2 μg/mL.^29^ Therefore, we assumed that a propofol concentration of 2.0 μg/mL is deep enough to suppress physical stimulation during emergence. In addition, further investigations into different target-controlled concentrations of propofol after LMA removal during emergence may provide important clues regarding clinic safety.

In summary, this investigation suggested that LMA removal in adults may be more successful in patients under deep anesthesia using TCI-administered propofol with a concentration of 2.0 μg/mL than in fully awake patients. Airway hyperreactivity was mild after propofol anesthesia.
